# Reviewer summary for Journal of Arrhythmia

**DOI:** 10.1002/joa3.12858

**Published:** 2023-06-13

The Editorial Board members of the Journal of Arrhythmia are grateful to the following reviewers who provided their expertise and knowledge to the journal.

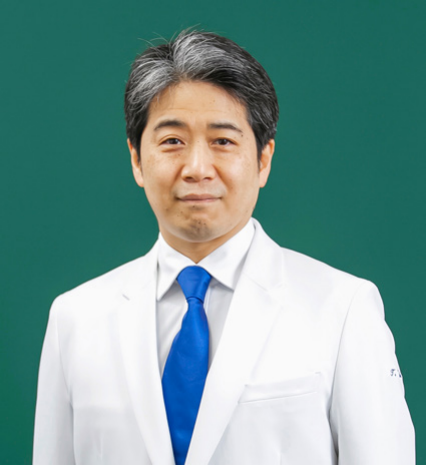

Noda, Takashi

nodatakashi@cardio.med.tohoku.ac.jp

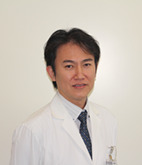

Yamashita, Seigo

seigoy722@yahoo.co.jp

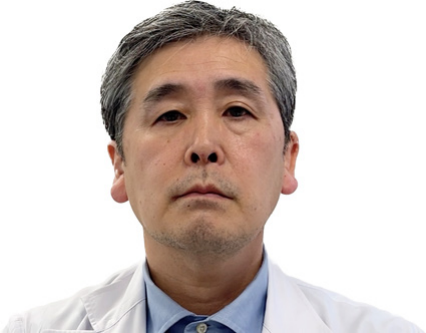

Yokoyama, Yasuhiro

yhy@me.com

Ching, Chi Keong

Ikeda, Yoshifumi

Inden, Yasuya

Morishima, Itsuro

Morita, Norishige

Fukamizu, Seiji

Fukaya, Hidehira

Sato, Toshiaki

Satomi, Kazuhiro

Sekihara, Takayuki

Yanagisawa, Satoshi

Asano, Taku

Kondo, Yusuke

Nagashima, Koichi

Nakahara, Shiro

Nishii, Nobuhiro

Oginosawa, Yasushi

Sasaki, Shingo

Tokuda, Michifumi

Yokoshiki, Hisashi

Arimoto, Takanori

Kawamura, Yuichiro

Ohe, Masatsugu

Takenaka, Sou

Yoshida, Kentaro

Hachiya, Hitoshi

Kabutoya, Tomoyuki

Kajiyama, Taketsugu

Kaneko, Yoshiaki

Kimura, Masaomi

Kumagai, Koji

Masuda, Masaharu

Miyanaga, Satoru

Naruse, Yoshihisa

Okumura, Yasuo

Imai, Katsuhiko

Maruyama, Mitsunori

Matsuo, Seiichiro

Minamiguchi, Hitoshi

Mine, Takanao

Mitsuhashi, Takeshi

Mukai, Yasushi

Nagase, Satoshi

Suzuki, Tsugutoshi

Yodogawa, Kenji

Aizawa, Yoshiyasu

Fujiu, Katsuhito

Fukunaga, Masato

Hasegawa, Kanae

Heeger, Christian

Higa, Satoshi

Ikeda, Kozue

Ishii, Yosuke

Kawakami, Hiroshi

Miyauchi, Yasushi

Nakai, Toshiko

Nodera, Minoru

Tokano, Takashi

Tsutsui, Kenta

Yagi, Tetsuo

Abe, Yoshihsa

Akima, Takashi

Chang, Shih‐Lin

Chinushi, Masaomi

Chung, Fa‐Po

Hashimoto, Kenichi

Hayashi, Kenshi

Hu, Yu‐Feng

Inoue, Koichi

Ishibashi, Kohei

Kohno, Ritsuko

Matsumoto, Kazuhisa

Miyazaki, Yuichiro

Nakajima, Ikutaro

Narita, Masataka

Shiga, Tsuyoshi

Shinohara, Tetsuji

Takahashi, Yoshihide

Takatsuki, Seiji

Tanaka, Yasuaki

Yada, Hirotaka

Yamaguchi, Takanori

Yoshiga, Yasuhiro

Yoshioka, Koichiro

Amaya, Naoki

An, Yoshimori

Bazoukis, George

Chang, Shih‐Lin

Ejima, Koichiro

Hayashi, Hidemori

Hayashi, Meiso

Irie, Tadanobu

Itoh, Taihei

Kamakura, Tsukasa

Komatsu, Yuki

Miyamoto, Koji

Miyazaki, Shinsuke

Nakamura, Kohki

Sasaki, Wataru

Sekiguchi, Yukio

Sumitomo, Naokata

Takahashi, Naohiko

Abe, Haruhiko

Aoki, Hisaaki

Fukuda, Koji

Horigome, Hitoshi

Kanzaki, Hideaki

Kataoka, Naoya

Kawamura, Iwanari

Kuroki, Kenji

Miyazaki, Aya

Mizutani, Yoshiaki

Mori, Hitoshi

Murakami, Masato

Nakasuka, Kosuke

Niwano, Shinichi

Rauber, Martin

Seo, Yoshihiro

Shizuta, Satoshi

Suzuki, Makoto

Usalp, Songül

Yamasaki, Hiro

Yoshimoto, Jun

Abe, Ichitaro

Arana‐Rueda, Eduardo

Chan, Yi‐Hsin

Chen, Wei‐Ta

Deshmukh, Abhishek

Foo, David

Furusho, Hiroshi

Harada, Masahide

Hayashi, Kentaro

Hayashi, Tatsuya

Imai, Yasushi

Kaitani, Kazuaki

Kamada, Hiroyuki

Karadeniz, Cem

Kawamura, Mitsuharu

Kobori, Atsushi

Kodani, Eitaro

Krasteva, Vessela

Kurita, Takashi

Kusa, Shigeki

Liao, Jo‐Nan

Lin, Jiunn‐Lee

Lo, Li‐Wei

Makiyama, Takeru

Matsumoto, Naoki

Matsushita, Kohei

Mizuno, Hiroya

Morita, Hiroshi

Nakajima, Kenzaburo

Nakatani, Yosuke

Nogami, Akihiko

Osanai, Hiroyuki

Sasaki, Takeshi

Sterns, Laurence

Takagi, Masahiko

Tan, Vern Hsen

Tanno, Kaoru

Teo, Wee‐Siong

Ueda, Akiko

Wakamiya, Akinori

Yagishita, Daigo

Yamagata, Kenichiro

Yamauchi, Yasuteru

Yoshida, Yukihiko

Aizawa, Yoshifusa

Akao, Masaharu

Cha, Myung‐Jin

Chanana, Shaurya

Chao, Tze‐Fan

Chiang, Chern‐En

Chiladakis, John

Dai, Shao‐Xing

Doi, Atsushi

Efremidis, Theodoros

Enjoji, Yoshihisa

Estner, Heidi L.

Goto, Kentaro

Hara, Hidehiko

Hasebe, Hideyuki

Hojo, Rintaro

Igarashi, Miyako

Ikeda, Takanori

Inaba, Osamu

Jeong, Young‐Hoon

Kanaoka, Koshiro

Kawada, Satoshi

Kumagai, Koichiro

Lee, Adam

Lever, Nigel

Lin, Lian‐Yu

Matsumoto, Katsumi

Mazzone, Patrizio

Mont, LluãS

Nagai, Toshiyuki

Nagase, Takahiko

Nagashima, Michio

Nakagawa, Koji

Nakano, Makoto

Ngarmukos, Tachapong

Ogawa, Masahiro

Ohno, Seiko

Okada, Atsushi

Osztheimer Md Phd, Istvan

Phrommintikul, Arintaya

Saplaouras, Athanasios

Sasano, Tetsuo

Shimizu, Akihiko

Shinohara, Masaya

Soeki, Takeshi

Takase, Bonpei

Takemoto, Masao

Thal, Sergio G.

Tilz, Roland

Tobiume, Takeshi

Toyohara, Keiko

Tsai, Chia‐Ti

Tsai, Chin‐Feng

Watanabe, Atsuyuki

Yagishita, Atsuhiko

Yamabe, Hiroshige

Yuniadi, Yoga

Zhou, Tao

